# P7C3-A20 neuroprotection is independent of Wallerian degeneration in primary neuronal culture

**DOI:** 10.1097/WNR.0000000000001146

**Published:** 2018-10-17

**Authors:** Ciaran S. Hill, David K. Menon, Michael P. Coleman

**Affiliations:** a John van Geest Centre for Brain Repair; b Department of Medicine, Division of Anaesthesia; c Department of Clinical Neurosciences, Wolfson Brain Imaging Centre, University of Cambridge, Cambridge, UK

**Keywords:** axon, P7C3-A20, protection, Wallerian degeneration

## Abstract

The antiapoptotic, neuroprotective compound P7C3-A20 reduces neurological deficits when administered to murine in-vivo models of traumatic brain injury. P7C3-A20 is thought to exert its activity through small-molecule activation of the enzyme nicotinamide phosphoribosyltransferase. This enzyme converts nicotinamide to nicotinamide mononucleotide, the precursor to nicotinamide adenine dinucleotide synthesis. Alterations to this bioenergetic pathway have been shown to induce Wallerian degeneration (WD) of the distal neurite following injury. This study aimed to establish whether P7C3-A20, through induction of nicotinamide phosphoribosyltransferase activity, would affect the rate of WD. The model systems used were dissociated primary cortical neurons, dissociated superior cervical ganglion neurons and superior cervical ganglion explants. P7C3-A20 failed to show any protection against WD induced by neurite transection or vincristine administration. Furthermore, there was a concentration-dependent neurotoxicity. These findings are important in understanding the mechanism by which P7C3-A20 mediates its effects – a key step before moving to human clinical trials.

## Introduction

**Fig. 1:**
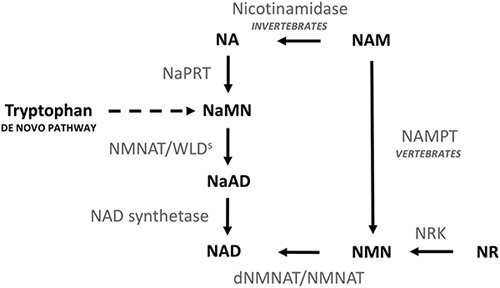
The nicotinamide adenine dinucleotide (NAD) synthesis pathway. The de novo NAD synthesis pathway generates NAD from the essential aromatic amino acid tryptophan. Nicotinic acid mononucleotide (NaMN) is converted to nicotinic acid adenine dinucleotide (NaAD) by nicotinamide mononucleotide adenylyltransferase (NMNAT). NaAD is then converted to NAD by NAD synthetase. There are three additional thee salvage pathways. Nicotinamide mononucleotide (NMN) is produced by nicotinamide phosphoribosyltransferase (NAMPT) from nicotinamide (NAM), and by nicotinamide riboside kinase (NRK) from nicotinamide riboside (NR). NMN is then converted to NAD by NMNAT. Alternatively, NAM is converted to nicotinic acid (NA) by nicotinamidase, and then NA is converted to NaMN by nicotinic acid phosphoribosyltransferase (NaPRT). P7C3-A20 is reported to acts as a small-molecule activator of the enzyme NAMPT within the bioenergetic NAD synthesis pathway.

In 2010 the antiapoptotic, neuroprotective aminopropyl carbazole agent P7C3 was discovered using a novel in-vivo screening approach 1. It has been proposed that the compound exerts its actions by activating the enzyme nicotinamide phosphoribosyltransferase (NAMPT). NAMPT is the rate-limiting enzyme in the nicotinamide adenine dinucleotide (NAD) salvage pathway that converts nicotinamide to nicotinamide mononucleotide (NMN), the precursor to NAD synthesis (Fig. 1). The NAD salvage pathway is important in Wallerian degeneration (WD), the active degeneration of an axon or neurite distal to a site of injury. P7C3, and one of its more active fluorinated enantiomers P7C3-A20, has shown promise as a therapeutic agent in a number of conditions that have been associated with WD. It was capable of blocking 1-methyl-4phenyl-1,2,3,4-tetrahydropyridine-induced death of dopaminergic substantia nigra cells in an adult mouse model of Parkinson’s disease 2. Its ability to protect spinal motor neurons from cell death was demonstrated both histologically and functionally in the G93A-SOD1 mouse model of amyotrophic lateral sclerosis 3. The potential for benefit of P7C3 derivatives in traumatic brain injury (TBI) was indicated by the findings that the analogue P7C3-S243, prevented retinal ganglion cell loss following experimental blast injury in mice 4. This was followed by a demonstration that P7C3-S243 also ameliorated behavioural deficits, including motor, learning, memory loss and axon loss in the CA1 stratum radiatum region of blast injury exposed mice 5. Furthermore, P7C3-A20 administration in a rat fluid-percussion injury model showed a reduction in contusion volume and improved sensorimotor and cognitive function 6.

The in-vivo phenotypic screening method by which the P7C3 compounds were discovered meant that further work has been required to elucidate their mode of action. Subsequent research has shown that P7C3-related compounds have some NAMPT-activating activity. NAMPT is an important enzyme in the WD pathway. However, the ability of NAMPT activation to protect against WD is not established and it has not been definitively shown that NAMPT activation is the means by which P7C3-based compounds exert their therapeutic action, or that they do not have other actions.

## Materials and methods

### Mouse origin and cell culture

C57BL/6 wild-type and SARM1^−/−^ mice were obtained from Babraham Institute (Babraham, UK). C57BL/6OlaHsd-Wlds (Wld^s^) mice, originally obtained from Harlan (Bicester, UK), were maintained as a separate colony at the Babraham Institute for 13 years. All animal work was performed in accordance with the 1986 Animals (Scientific Procedures) Act under project licence PPL 70/7620. All superior cervical ganglion explant (SCGe) and dissociated superior cervical ganglion (dSCG) cultures were obtained from humanely killed postnatal day 0 (P0) to P2 mice and were prepared as described previously 7,8. Cells were cultured for 7 days *in vitro* before experimentation. SCGe were plated on 35 mm Nunc cell culture dishes (Thermo-Fisher Scientific, Waltham, Massachusetts, USA), and dSCGs were plated on low 35 mm μ-dishes (ibidi, Martinsried, Germany). Primary cortical neuronal cells were extracted by dissecting out a section of cortical mantle, trypsinizing and dissociating the neurons before separating by centrifugation. Viability was confirmed to be greater than 95%. The cells were then plated and cultured on low 35 mm μ-dishes (ibidi) for 7 days *in vitro* before experimentation. In all cases, dishes were coated with poly-l-lysine (25 μg/ml; Sigma, St. Louis, Missouri, USA), then laminin (2 μg/cm^2^; Sigma). All cultures were maintained in an incubator at a constant 37°C with 5% CO_2._

### P7C3-A20

The compound P7C3-A20 (cat no. 2850) was sourced from Cambridge Bioscience (Cambridge, UK) and supplied by Biovision (Milpitas, California, USA) and prepared and used according to manufacturer’s instructions.

### Transection and imaging

Transection injury of SCGes was undertaken by a single linear incision made 2 mm from the cell mass with a #10 blade scalpel (Swann-Morton, Sheffield, UK). All images of SCG and primary cortical neuron (PCN) cultures were taken on an Olympus IX81 inverted microscope system (Olympus, Shinjuku, Tokyo, Japan) using ×20 magnification and phase-contrast imaging.

### Neurite degeneration index

The neurite degeneration index (NDI) is an established quantitative measure of neurite degeneration (ND) 9. Images of the same, or similar, the field of neurites are obtained at different time points or treatment conditions at a controlled level of magnification (×20) and illumination. In the case of SCGe the imaged field is typically 5 mm from the main explant mass where the density of the neurites allows clear visualization of morphological change. The NDI is calculated by an Image J (University of Wisconsin, Wisconsin, USA) plug-in algorithm that is operator independent. The output is a unitless number from 0 to 1 based on the degree of neurite continuity or fragmentation. Most healthy neurites score around 0.1–0.3, whereas a profoundly degenerated field would be in the range of 0.6–0.9. In the case of complete degeneration, with or without detachment, a score of 0.9 is assigned 9.

### NAD/NADH-Glo assay

Levels of NAD were measured using the NAD/NADH-Glo assay (Promega, Madison, Wisconsin, USA). The assay was performed according to the manufacturer’s protocol. The assay contains a cycling reductase enzyme that reduces a proluciferin reductase substrate to luciferin in the presence of NADH. Luminescence signals were measured by PHERAstar plate reader (BMG Labtech, Ortenberg, Germany).

### Statistics

Data are expressed as a mean value with error bars representing SEM unless otherwise stated. A number of repeats and replicates and statistical tests are detailed in each figure legend. A *P* value of 0.05 or less was considered significant for any data set. Asterisks represent the statistical significance as follows: *P≤0.05, **P≤0.01, ***P≤0.001, ****P≤0.0001, *P*<0.05 (NS). All statistical tests and graphs were generated with GraphPad Prism Software, version 7.0 (GraphPad Software Inc., La Jolla, USA).

## Results

**Fig. 2:**
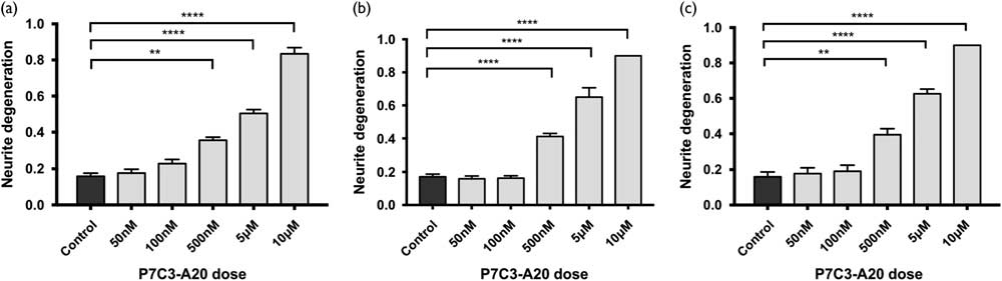
P7C3-A20 triggers concentration-dependent spontaneous neurite degeneration in a variety of primary neuronal cell types. Neurite degeneration (ND) was induced in response to various concentrations of P7C3-A20 for 24 h in (a) dissociated superior cervical ganglion cells, (b) superior cervical ganglion explant and (c) primary cortical neuron cell cultures, in a range of concentrations. Neurite degeneration index was calculated at the time of treatment and at 24 h afterward. ND shows a concentration-dependent increase with P7C3-A20 administration across all neuronal cell types. The treatment concentration for subsequent experiments was determined as the maximum concentration at which ND did not occur within this timeframe (100 nΜ in all cell types). *n*=12 independent repeats per condition. **P≤0.01, ***P≤0.001, ****P≤0.0001. Error bars show SEM. Statistics: one-way analysis of variance.

**Fig. 3:**
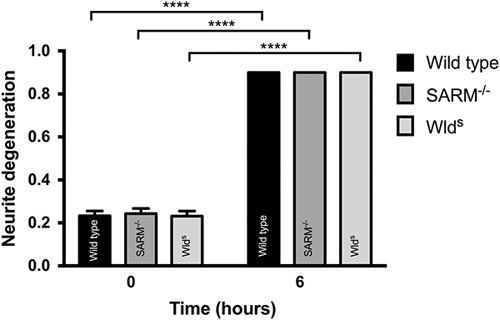
Removal of SARM1 or expression of Wld^s^ in primary cortical neuron (PCN) and superior cervical ganglion explant (SCGe) cultures did not protect against neurite degeneration induced by P7C3-A20. PCNs and SCGe cultured from wild-type, transgenic SARM1^−/−^, and WLD^s^ mice were treated with 10 μM of P7C3-A20 and neurite degeneration measured 6 h later. In all conditions, there was complete neurite degeneration and subsequent detachment of the cells. *n*=9 independent repeats. ****P≤0.0001. Error bars show SEM. Statistics: Student’s *t*-test.

To select an appropriate concentration of P7C3-A20 to test in WD, we first assessed for toxicity over a wide concentration range (50 nm to 10 μM) in a variety of neuronal cell types (Fig. 2). P7C3-A20 induces spontaneous ND, as measured by the NDI 6, in a concentration-dependent manner in murine SCGe, SCGd and PCN cultures but concentrations of 100 nM or below were found to cause no toxicity at the 24-h timepoint. The ND at concentrations above 100 nM might follow a Wallerian-like mechanism following activation of NAMPT leading to overproduction of NMN. To test this possibility, we asked whether SCGe cultures from SARM1^−/−^ and Wld^s^ mice showed any less ND at a 10 μM concentration of P7C3-A20, a concentration that we demonstrated induced ND within 6 h (Fig. 3). In both SARM1^−/−^ and Wld^s^ cultures, the degeneration was rapid and comparable to wild-type cultures. This suggests the mechanism of degeneration at high concentrations of P7C3-A20 is independent of the WD pathway.

**Fig. 4:**
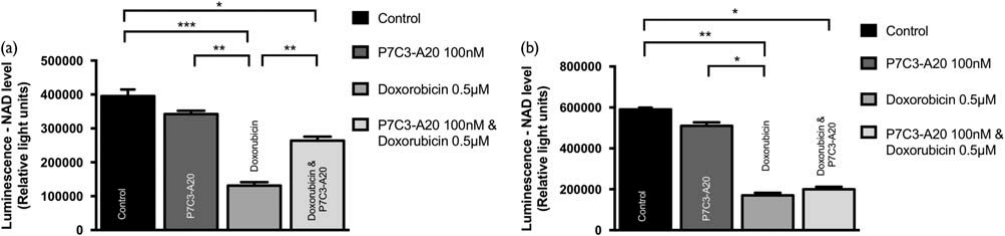
Doxorubicin induces nicotinamide adenine dinucleotide (NAD) depletion, which is rescued by P7C3-A20 in primary cortical neuron (PCN) cultures but not in superior cervical ganglion explant (SCGe). (a) PCNs were treated with either DMSO (as control), 100 nΜ of P7C3-A20, 500 nM of doxorubicin, or a combination of 100 nΜ P7C3-A20 and 500 nM doxorubicin, and incubated for 48 h. The NAD levels were then analysed using a NAD/NADH-Glo luciferin-based assay (Promega). Luminescence signals were measured and quantified by a PHERAstar plate reader. Doxorubicin depleted NAD levels in the PCNs and this was partially rescued by P7C3-A20. (b) The experiment was repeated with an SCGe culture but no significant rescue of NAD levels was seen. *n*=9 independent repeats. *P≤0.05, **P≤0.01, ***P≤0.001. Error bars show SEM. Statistics: one-way analysis of variance.

We then tested whether 100 nM P7C3-A20 was able to rescue NAD depletion caused by doxorubicin in these neuronal types, as was previously reported for human bone osteosarcoma epithelial (U2OS) cells 10 (Fig. 4). Doxorubicin-treated PCN and SCGe cultures showed a reduction in NAD levels as measured by a luciferin based (NAD+NADH) assay. This was from an average of 395 485 to 131 406 relative light units in PCN (66.8% reduction, *P*≤0.001), and 590892 to 170363 relative light units in SCGe (71.2% reduction, *P*<0.01). When doxorubicin-exposed cells were co-treated with P7C3-A20, there was a partial rescue in NAD levels in PCN cultures with an average output of 263 886 relative light units; a 34.4% greater maintenance of NAD levels than seen in untreated cultures (*P*≤0.001). However, P7C3-A20 did not rescue the NAD level in SCGs treated with doxorubicin; these had an average output of 200 318 relative light units. This was not significantly different than the average 170 363 light units seen in SCGs treated with doxorubicin alone. While we cannot rule out loss or degradation of NAD from doxorubicin-treated SCGs, this failure to rescue the NAD levels in SCGs suggests the ability of P7C3-A20 to activate NAMPT may not be conserved across all neuronal cell types.

**Fig. 5:**
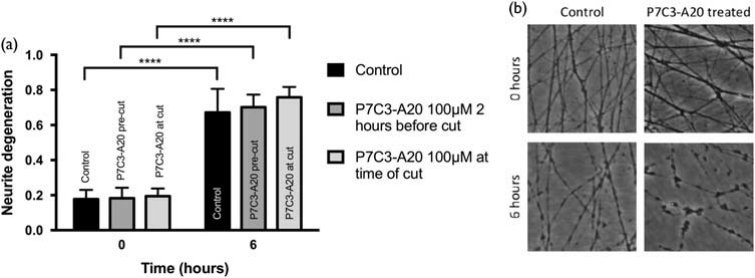
P7C3-A20 failed to delay Wallerian degeneration in superior cervical ganglion explant (SCGe) following transection. (a) The SCGes were 5-7 DIV, and either pretreated with P7C3-A20 1 h before cut or at the time of cut. DMSO was used as a control at the time of cut. There was a comparable degree of degeneration in all conditions at a 6 h timepoint. (b) Representative images of the SCGe cultures are shown at the time of cut and 6 h later for the control and the P7C3-A20 treated condition. *n*=8 independent repeats. ****P≤0.0001. Error bars show SEM. Statistics: Student’s *t*-test.

Neurite transection of untreated wild-type SCGe cultures resulted in a progressively increased ND at 6 h – with a precut degeneration index average of 0.70 and a 6 h post cut index of 0.67 (*P*≤0.0001) (Fig. 5). When pretreated with P7C3-A20 2 h before transection, or immediately before the transection, there was no significant difference in the degree of WD between the untreated and treated cultures (average of 0.70 for pretreated and 0.76 for those treated at time of cut), hence indicating that the reported neuroprotective effects of P7C3-A20 are unlikely to be mediated by a delay in WD.

**Fig. 6:**
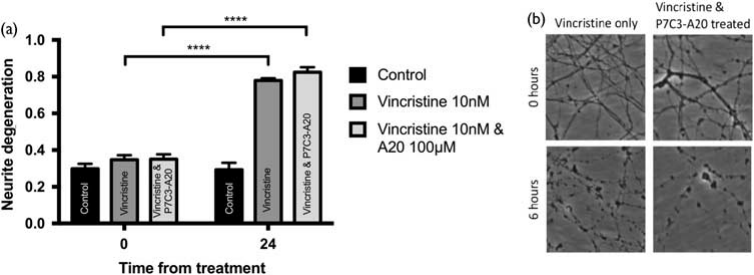
P7C3-A20 treatment did not prevent vincristine-induced Wallerian degeneration in primary cortical neuron (PCN) cells. (a) Dissociated primary neuronal cortical cells were treated with DMSO (as control), 10 nM of vincristine, or 10 nM of vincristine and 100 nΜ of P7C3-A20. The addition of P7C3-A20 failed to prevent vincristine-induced Wallerian degeneration. (b) Representative images of the PCN cultures are shown at the time of vincristine treatment and 6 h later for the control and the P7C3-A20 co-treated condition. *n*=8 independent repeats. ****P≤0.0001. Error bars show SEM. Statistics: Student’s *t*-test.

In dispersed PCN cultures, neurite length and growth patterns make their physical transection unreliable, so Wallerian-like degeneration was induced with a 100-nM concentration of vincristine (Fig. 6). Baseline ND scores in untreated control PCN were an average of 0.3. Six hours after treatment with vincristine these rose to 0.78 (*P*≤0.0001). Unlike Wld^s^ and knockout of SARM1 11,12, co-treatment of cultures with P7C3-A20 in addition to vincristine did not prevent ND with an average NDI of 0.82.

## Discussion

The drug P7C3-A20 has shown promise in various animal models of neurological disease, including TBI 6, chronic visual deficits 4, Parkinson’s disease 2 and amyotrophic lateral sclerosis 13. It has also been suggested as a possible therapeutic agent for other neurodegenerative diseases including Alzheimer’s disease, and as a preserver of cognitive capacity in aging 1,13. P7C3-A20 has been shown to enhance neuron formation in the subgranular zone of the dentate gyrus of adult mice and protected against apoptotic loss of newborn hippocampal neurons in neuronal PAS domain protein 3 knockout mice 1.

The in-vivo phenotypic screen by which the P7C3 compounds were discovered meant that further work was required to elucidate their mode of action. This involved triggering the death of human osteosarcoma (U2OS) cells using eight toxic compounds and testing whether P7C3 could rescue any of these. In this way, doxorubicin toxicity was found to cause P7C3 sensitive cell death. Doxorubicin is a commonly used chemotherapeutic agent that acts through a mechanism involving intracellular depletion of NAD. NAD was partially restored in a concentration-dependent manner by U2OS cell treatment with P7C3 enantiomers. A photocrosslinking approach followed by click chemistry with Alexa 532 dye then showed that P7C3 bound to NAMPT. These findings suggested that P7C3 activity might be related to NAMPT activation and related NAD preservation. This theory is particularly intriguing as NAMPT and NAD are key molecular players in WD, the process by which an axon or neurite degenerates distal to a site of injury. The active degeneration of WD occurs in both central and peripheral neurons, including PCNs, and may be an important component of several pathological entities including many neurodegenerative disorders, neuropathies and TBI 14. WD is now recognized as an active cell-autonomous death pathway.

To progress from animal models to human trials, it is important to have a thorough understanding of the mechanistic effects of a drug. This study was undertaken to further characterize the mechanism of P7C3-A20 action and to explore the proposition that its effects are because of NAMPT activation leading to protection against WD. Several key steps in the WD pathway have been identified including loss of the enzyme nicotinamide mononucleotide adenylyltransferase 2 (NMNAT2), for example, in distal neurites because of interrupted delivery from the soma following transection injury or transport block 15,16. Ordinarily, NMNAT2 is produced in the cell soma and shuttled by kinesin-mediated axonal transport to distal neurites. Lack of NMNAT2 results in an inability to convert NMN to NAD. This leads to a rise in NMN and fall in NAD levels in the distal axon, and is then followed by a step dependent on sterile-alpha and TIR motif containing protein 1 (SARM1) molecule and subsequent calcium-mediated axonal fragmentation 17. SARM1 is a Toll-like receptor adaptor protein that is a major player in WD when it is knocked out there the result is delayed WD. A related spontaneous slow-WD mutation ‘Wld^s^’ phenotype exists where animals have a long-acting NMNAT2 like protein ‘WLD^s^’ that can substitute for lost NMNAT2 and delay the active WD process. Another important enzyme in the WD pathways is NAMPT. The role of NAMPT in WD can be demonstrated through the use of the NAMPT inhibitor FK866; this blocks the conversion of nicotinamide to NMN. When murine SCGe neurons were pretreated with FK866 up to a day before, or up to 3 h after, transection injury, they showed a delayed WD phenotype for 24–48 h. However, after 96 h even uninjured neurites spontaneously degenerated 9. The initial delay in WD may have been due to a reduction in NMN accumulation as the addition of exogenous NMN could revert protection by FK866. Their eventual degeneration may be caused by NAD depletion and cellular energetic failure.

P7C3-A20 has previously been shown to increase NAD levels in U2OS cells; following this, we found it was able to increase NAD levels in selected primary neuronal cell types. Furthermore, no protection against WD was found in any cell type, regardless of whether the injury was physical transection or by vincristine. Vincristine is a chemotherapeutic drug that causes a length-dependent axon degeneration 18. This degeneration is blocked by Wld^s^ or removal of SARM1 1,11,19. Hence, vincristine is an example of an exogenous chemical agent that can be used to trigger Wallerian-like degeneration without the requirement for a physical injury.

The ability of NAMPT to catalyze the metabolism of nicotinamide to NMN raises intriguing questions about how P7C3 may influence WD. As long as NMNAT2 is present in the axon, it will use NMN to synthesize NAD, but after axon injury, NMNAT2 is rapidly degraded 10,11 and NMN accumulates 6. Increasing NAMPT activity after cutting axons would be expected to lead to faster accumulation of NMN and thus might increase the rate of degeneration. In studies where NAMPT expression was instead reported to be protective, its expression was increased before axons were cut 20. The ability of NAMPT inhibition to delay WD contrasts with the proposed action of P7C3-A20 to block cell death mechanisms through augmenting NAMPT activity. To resolve this, we tested the effect of P7C3-A20 on the survival of both injured and intact axons. We found that P7C3-A20 was unable to prevent doxorubicin-induced death or to delay WD in either primary neuronal culture or superior cervical ganglion cell cultures. Therefore, any therapeutic benefits of the compound may act through different mechanistic pathways.

Our findings did not support a proposed mechanism of P7C3-A20 as a NAMPT activator leading to protection against WD. This suggested that P7C3-A20’s mechanism of action needs further investigation. Its failure to protect against WD suggests that it might not be an appropriate agent to treat diseases that have a large WD-like component, for example, chronic inflammatory peripheral neuropathy, glaucoma and some forms of hereditary motor sensory neuropathy.

A further finding was the concentration-related toxicity of P7C3-A20. It has previously been reported in rodent models to have a wide-therapeutic index 10. In our study, we found that there was a marked concentration-dependent toxicity in all primary neuronal cultures. The failure of Wld^s^ and SARM1 knockout transgenics to protect against this suggests that it is due to a mechanism independent of WD. This toxicity needs to be better understood, both in its mechanism, in an in-vitro setting and in the design of any human clinical trials.

## Conclusion

The antiapoptotic, neuroprotective agent P7C3-A20 caused ND at concentrations of 500 nM or greater in dSCG, SCGe and PCN. At concentrations of 100 nM, P7C3-A20 was able to partially rescue doxorubicin-induced NAD depletion in PCN, but not in SCGe. P7C3-A20 failed to delay WD induced by transection in SCGe, or by vincristine in dissociated PCN. These findings are significant in understanding the consequences of exposing neurons to various concentrations of P7C3-A20 and identifying the mechanisms by which the compound exerts its action. Additional investigation of the mechanistic action of P7C3-A20 is required to build the necessary foundation for potential human studies.
